# How the Discovery of the CD4/CD8-p56^lck^ Complexes Changed Immunology and Immunotherapy

**DOI:** 10.3389/fcell.2021.626095

**Published:** 2021-03-15

**Authors:** Christopher E. Rudd

**Affiliations:** ^1^Division of Immunology-Oncology, Centre de Recherche Hôpital Maisonneuve-Rosemont (CR-HMR), Montreal, QC, Canada; ^2^Department of Microbiology, Infection and Immunology, Faculty of Medicine, Universite de Montreal, Montreal, QC, Canada; ^3^Division of Experimental Medicine, Department of Medicine, McGill University Health Center, McGill University, Montreal, QC, Canada

**Keywords:** p56^lck^ tyrosine kinase, CD4, CD8, tyrosine phosphorylation, chimeric antigen receptor, immunotherapy, T-cell signaling paradigm, protein-tyrosine activation cascade

## Abstract

The past 25 years have seen enormous progress in uncovering the receptors and signaling mechanisms on T-cells that activate their various effecter functions. Until the late 1980s, most studies on T-cells had focused on the influx of calcium and the levels of cAMP/GMP in T-cells. My laboratory then uncovered the interaction of CD4 and CD8 co-receptors with the protein-tyrosine kinase p56^lck^ which are now widely accepted as the initiators of the tyrosine phosphorylation cascade leading to T-cell activation. The finding explained how immune recognition receptors expressed by many immune cells, which lack intrinsic catalytic activity, can transduce activation signals via non-covalent association with non-receptor tyrosine kinases. The discovery also established the concept that a protein tyrosine phosphorylation cascade operated in T-cells. In this vein, we and others then showed that the CD4- and CD8-p56^lck^ complexes phosphorylate the TCR complexes which led to the identification of other protein-tyrosine kinases such as ZAP-70 and an array of substrates that are now central to studies in T-cell immunity. Other receptors such as B-cell receptor, Fc receptors and others were also subsequently found to use *src* kinases to control cell growth. In T-cells, p56^lck^ driven phosphorylation targets include co-receptors such as CD28 and CTLA-4 and immune cell-specific adaptor proteins such as LAT and SLP-76 which act to integrate signals proximal to surface receptors. CD4/CD8-p56^lck^ regulated events in T-cells include intracellular calcium mobilization, integrin activation and the induction of transcription factors for gene expression. Lastly, the identification of the targets of p56^lck^ in the TCR and CD28 provided the framework for the development of chimeric antigen receptor (CAR) therapy in the treatment of cancer. In this review, I outline a history of the development of events that led to the development of the “TCR signaling paradigm” and its implications to immunology and immunotherapy.

## Introduction

The last decades have witnessed major advances in the identification of the receptors and signaling pathways that control the activation and differentiation of T-cells. Early work in understanding the key signaling events involved the demonstration that anti-CD3 antibodies could increase intracellular calcium (Ca^2+^) levels as detected by the Indo-1 indicator dye (Tsien et al., 1982). Other pathways involved the identification of oscillations in cAMP/cGMP, the activation of phospholipase C (PLC) which was known to hydrolyze phosphatidylinositol-4,5-bisphosphate (PIP2) into the Ca^2+^-mobilizing second messenger, inositol-1,4,5-trisphosphate (IP3) and diacylglycerol (DAG) (Imboden and Stobo, 1985). DAG is a physiological activator of protein kinase C (PKC). Oscillations in calcium were later shown to be essential to the activation of the transcription factor nuclear factor of activated T-cells (NFAT) (Shaw et al., 1988; Jain et al., 1992).

## CD4/CD8-p56^lck^ and the Initiation of TCR Signaling

Despite this important work, a critical missing area was the possible involvement of protein tyrosine phosphorylation in T-cells. Emerging data had underscored the importance of this type of phosphorylation in regulating multiple events in other mammalian cells. Most phosphorylation occurs on serine and threonine with <1% on tyrosine residues. Tony Hunter had described phosphorylation on tyrosine residues in the late 1970s, working on middle T-antigen (Eckhart et al., 1979). Transmembrane receptors such as the platelet-derived growth factor receptor (PDGF-R) and the insulin receptor were then found to have intrinsic protein-tyrosine kinase domains in their cytoplasmic tails (Rudd, 1990; Hunter, 2007). However, another family of soluble protein-tyrosine kinases had also been defined with the prototype pp60^src^. Notably, a truncated form of the kinase termed pp60^v−src^ had been identified in the *Rous sarcoma virus* which acted as an oncogene (Parker et al., 1981). Michael Bishop and Harold Varmus had won the 1989 Nobel Prize for showing that the oncogene in the virus was an altered version of a gene derived from the normal cellular gene of normal cells. However, the cellular homolog pp60^src^ had no apparent function in mammalian cells. A role for *src* family members in normal cell function had been unclear. The *src* family of non-receptor tyrosine kinases (SFKs) include Src, Fyn, Yes, Lck, Hck, Blk, Fgr, Lyn, and Yrk (Neet and Hunter, 1996; Serfas and Tyner, 2003). Src, Yes, Lyn, and Fyn are widely expressed in cells, while Blk, Fgr, Hck, and Lck are expressed primarily in hematopoietic cells (Thomas and Brugge, 1997). T cells express predominantly Lck and Fyn that include an alternatively spliced isoform of Fyn termed Fyn^T^.

In immunology, there was a major gap in knowing whether protein-tyrosine kinases, or a potential phosphorylation cascade operated in T-cells and other immune cells. There were no known surface receptors with endogenous protein-kinase domains connected to the antigen-receptor (TCR/CD3 complex) and little evidence of tyrosine phosphorylation in immune cells. The main evidence came from studies on LSTRA cells, T-cell lymphoma transformed by the Moloney Murine Leukemia Virus that showed elevated tyrosine phosphorylation of intracellular proteins (Casnellie et al., 1982; Gacon et al., 1982; Voronova et al., 1984). However, it was unclear whether this was an anomaly and whether receptors on normal T-cells engage tyrosine kinases to evoke a phosphorylation cascade. The lab of Larry Samelson and Richard Klausner provided some of the first hints by showing that a p21 chain associated with the T cell antigen receptor underwent tyrosine phosphorylation of 294 hybridoma T-cells (Samelson et al., 1986b).

The central problem was that neither the TCR itself nor its associated CD3 γ/ε, δ/ε, or ζ chains showed sequence homology with known protein-tyrosine kinases. Given this situation, it seemed a reasonable possibility to us that the TCR might be coupled to an unidentified transmembrane tyrosine kinase receptor, an activator of a kinase protein tyrosine kinase, or in some unusual manner, might bind to a protein-tyrosine kinase. Our initial studies initially showed little endogenous kinase activity co-precipitated with the anti-CD3 precipitated TCR complex in auto-phosphorylation kinase assays. This observation shifted our attention to the co-receptors CD4 and CD8, which had recently been shown to bind to non-polymorphic regions of the major histocompatibility complex (MHC) (Meuer et al., 1982). For example, the α chain of the CD8 complex binds to HLA's α2 and α3 domains of MHC class 1 antigens (Gao et al., 1997). We envisioned that a situation where a kinase associated with CD4 and CD8 might be brought into physical proximity with the TCR complex for its phosphorylation.

From the outset of our work in 1986, we found that immune precipitates of CD4 and CD8 possessed an unusually high level of endogenous tyrosine kinase activity that was not observed in the precipitates of other receptors. Further, in addition to the phosphorylation of the exogenously added substrate, enolase, we observed a well-labeled band in the 56–65 Kd range in anti-CD4 and CD8 precipitates that was labeled on tyrosine residues (Rudd et al., 1988; Barber et al., 1989). Two other bands in the 30–35 Kd and 75–80 Kd range were also labeled in the anti-CD4 and CD8 precipitates (Rudd et al., 1988; Barber et al., 1989). None of these bands corresponded to CD4 or CD8 indicating that the co-receptors themselves were unlikely to be substrates of the endogenous co-precipitated kinase.

Independent work on pp60^src^ had shown that *src*-related kinases could phosphorylate themselves in a process termed auto-phosphorylation. This occurs when a kinase's active site catalyzes its own phosphorylation (cis autophosphorylation), or when a kinase provides the active site of an adjacent kinase (trans autophosphorylation). It did not escape our notice that the band at 55–65 kd was of a similar size as pp60^c−src^, although src was poorly expressed in T-cells. Perhaps a related kinase might be phosphorylating itself in precipitates, and perhaps it was immune cell-specific mirroring the cell-specific nature of receptors on the surface of immune cells. It may seem self-evident now, with the available information, but at the time this was a rather grand conceptional jump. In this context, a protein at 56 Kd, originally termed LSTRA protein-tyrosine kinase had been seen in LSTRA lymphoma T-cells by the labs of Bart Sefton and Edwin Krebs (Casnellie et al., 1982; Gacon et al., 1982; Voronova et al., 1984). The kinase was subsequently cloned by Jamey Marth in the lab of Roger Perlmutter [encoded by a genetic locus defined as lsk^T^] and found to be a T-cell-specific member of the pp60^src^ family, LCK or p56^lck^ (Marth et al., 1985). However, as in the case of the parental kinase pp60^src^, no function for p56^lck^ had been identified in normal T-cells. The idea that *src* kinases could in some manner interact with surface receptors, rather than interacting solely with intracellular components such as middle T-antigen, had not been established.

**Figure 1:**
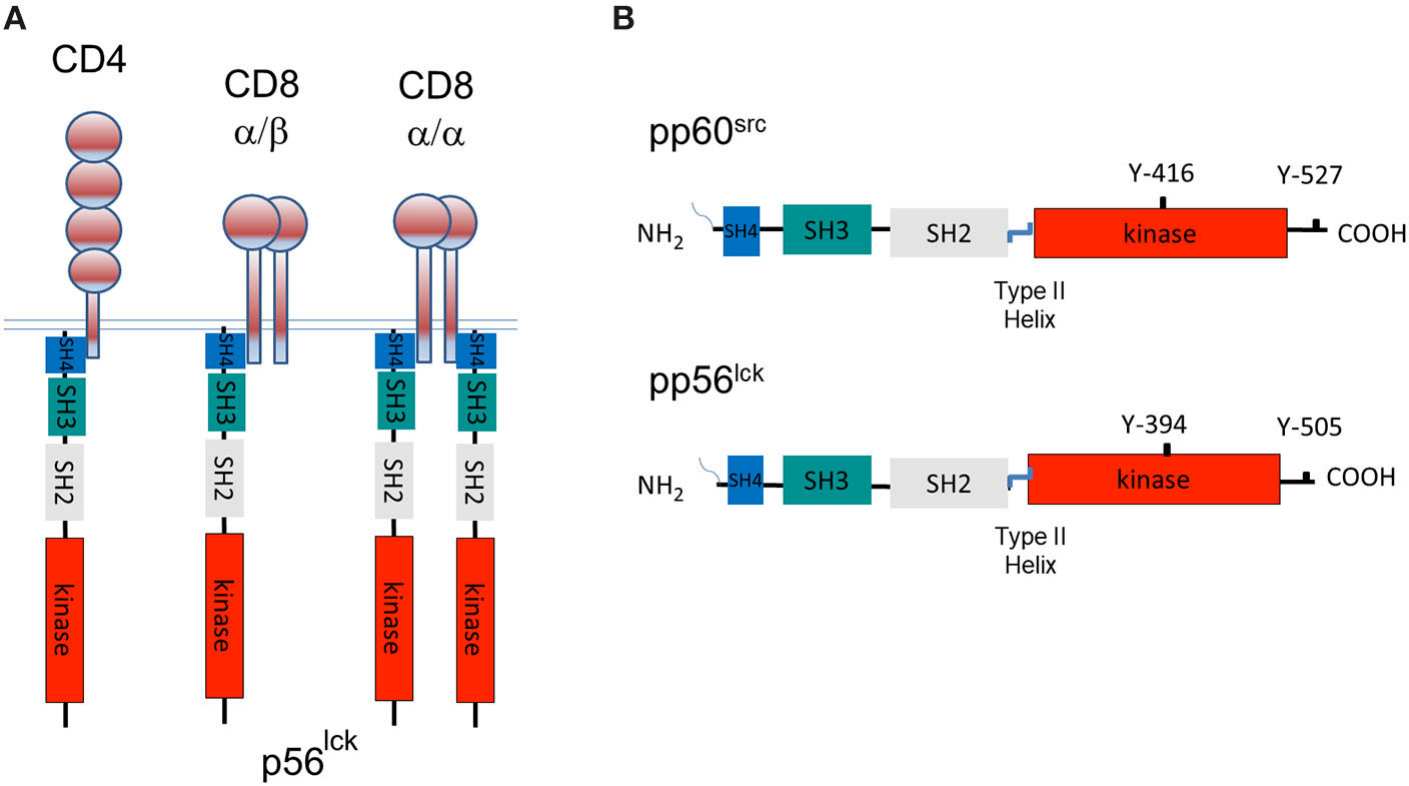
A tale of three CD4 and CD8-p56^lck^ complexes and the structure of pp60^src^ and p56^lck^. **(A)** The model of three CD4 and CD8-p56^lck^ complexes in T-cells. CD8 is expressed as a CD8α homodimer as well as a CD8α/β heterodimer. p56^lck^ binds to the α subunit but not the β subunit. CD8α homodimer has two p56^lck^ bound molecules and the CD8α/β heterodimer has a single p56^lck^ bound. CD4 binds to p56^lck^ in a monomeric form. **(B)** Structure of pp60^src^ and p56^lck^. p56^lck^ is an immune cell-enriched member of the pp60^src^ family of protein-tyrosine kinases. p56^lck^ is myristoylated and palmitoylated at the N-terminus, while Src lacks palmitoylation sites. This region is followed by poorly conserved unique SH4 region which in the case of p56lck binds to the cytoplasmic tails of CD4 and CD8, an SH3 domain that binds to proline-rich residues, an SH2 domain that binds to specific sites that are tyrosine phosphorylated, an SH2-kinase linker region, an SH1 kinase domain followed by a C-terminal negative regulatory region. The C-terminal tail has an inhibitory Y-527 site when phosphorylated, in the case of pp60^src^ and a Y-505 site in p56^lck^. pp60^src^ and p56^lck^ also possess an auto-phosphorylation site in the kinase domain of each kinase corresponding to Y-416 in the case of pp60^src^ and Y-394 in the case of p56^lck^.

Using an anti-p56^lck^ sera from Jim Trevillyan at the University of Texas, we showed that it reacted with our 56Kd protein that had been labeled *in vitro* kinase assays using a combination of blotting and re-precipitation analysis (Rudd et al., 1988; Barber et al., 1989). This clearly showed that the CD4 and CD8 receptors interacted with the *src* family member called p56^lck^. In our original paper, we stated: “the association appears to represent the only known case of an association between a receptor on the surface of T cells and a member of a family of intracellular mediators with an established ability to activate and transform cells.” The fact that both CD4 and CD8 bound to p56^lck^ was consistent with their similar, but complementary roles in binding to non-polymorphic regions of MHC class II and class 1 antigens, respectively. CD4 binds to p56^lck^ in a monomeric form, although in certain contexts, the receptor may form dimers or multimers (Lynch et al., 1999; Matthias et al., 2002; Figure 1A). By contrast, CD8 exists as a α/β heterodimer or a α/α homodimer within which the p56^lck^ binds to the CD8α subunit. The homodimer can recruit two p56^lck^ molecules, while the CD8α/β heterodimer binds a single p56^lck^ (Figure 1A).

The CD4 and CD8-p56^lck^ complexes were the first examples of a protein-tyrosine kinase to associate with a surface receptor. They were also the first case of an interaction with an SFK and explained how receptors that lack intrinsic catalytic activity could transduce activation signals. The interaction provided a mechanism by which the antigen receptor could induce a possible tyrosine phosphorylation cascade in T-cells and put the focus on p56^lck^ as the central player of T-cell activation, some of which is receptor associated and the rest of which exists in a receptor-free form in cells.

Our original submitted paper languished for over a year with Nature from 1986 to 1987, at which time we decided to re-submit to PNAS for publication and to file patents, which were filed and granted several years later (Nos. 5,250,431, 1993, US5432076; EP0347143A2, 1988). I also began to discuss our unpublished findings openly with colleagues at the Dana-Farber Cancer Institute which led to a contact from Andre Veillette in the lab of Joseph Bolen at the National Institutes of Health. After some discussion, they agreed to collaborate showing the presence of the CD4 and CD8-p56^lck^ complexes in mouse cells (Veillette et al., 1988). This collaborative work was very important and helpful to us, given that, at the time, my group was comprised of a young technician and myself, without an established reputation in the field of protein-tyrosine kinases. The work in our first paper was supported by the Cancer Research Institute (NY), an organization whose founding was based on the work of Dr. William B. Coley in the late 1800s to treat cancer patients with immunotherapy. We were gratified that our CD4 and CD8-p56^lck^ complexes as initiators of the activation cascade in human T-cells are the same signal mediators that stimulate T-cells to react and kill tumors in immunotherapy. Our first paper was recognized as “*Pillars of Immunology*” paper by the American Association of Immunologists together with a paper from our collaborators in the Bolen lab (Rudd et al., 2010; Veillette et al., 2010).

CD4 and CD8-p56^lck^ complexes became models for how other immune receptors employ SFKs in immune cell activation. Lyn and Fyn were subsequently found to associate with the Igα/Igβ heterodimer subunits of the B cell receptor in B-cells (Gauld and Cambier, 2004), Src and Lyn to the Fc receptor (FCR) (Wu et al., 2001) and Fyn and Lyn to the glycoprotein VI (GPVI)-FcR gamma-chain complex, a key receptor for collagen on platelets (Suzuki-Inoue et al., 2002). In fact, a single Lyn single molecule may be sufficient to initiate phosphorylation of multiple aggregated high-affinity IgE receptors (Wofsy et al., 1999). Further, pp60^Src^ is activated by binding the integrin β cytoplasmic domain (Arias-Salgado et al., 2003), while in T-cells, p59^fyn^, and p56^lck^ associates, albeit with lower stoichiometry, with the CD3 subunits of the TCR receptor (Hartl et al., 2020). p56^lck^ was also been found to associate with the co-receptor CD28 by using its SH2 domain to bind to a phospho-specific site (Kong et al., 2011).

With an emphasis placed on p56^lck^, it was subsequently ablated in mice and found to be needed for the early and late stages of thymic differentiation (using proximal and distal Lck promoters) (Teh et al., 1991), naive T cell survival (Seddon and Zamoyska, 2002), and T-cell activation. Lck/Fyn double deficient mice show a 3 stage (DN3) block in the thymus which requires pre-TCR signaling (Liao et al., 1997). Similarly, B-cells require Lyn kinase activity for B-cell receptor phosphorylation and function (Fujimoto et al., 1999). Likewise, macrophages lacking the Hck and Lyn are defective in IgG-mediated phagocytosis (Fitzer-Attas et al., 2000). Other examples exist.

In the field of cancer biology, as mentioned, previous seminal work had documented how truncated forms of pp60^v−src^ transformed cells; however, a role for non-oncogenic src-related kinases had been missing. Other non-lymphoid surface receptors, such as the platelet-derived growth factor receptor (PDGF-R) were eventually also shown to bind and generate signals via SFKs (Thomas and Brugge, 1997; Rudd, 1999).

Lastly, our studies impinged on the field of acquired immunodeficiency syndrome (AIDS) and the human immunodeficiency virus (HIV-1), being the first example of a mediator to associate with the HIV-1 receptor, CD4 (Rudd et al., 1988). p56^lck^ and its binding to CD4 were later shown to provide signals that regulate HIV-1 transcription in T-cells (Tremblay et al., 1994). HIV-1 induced apoptosis is accelerated by interaction of CD4 with p56^lck^ (Corbeil et al., 1996).

## CD4/CD8-p56^lck^ and Phosphorylation of the TCR Complex

The CD4/CD8-p56^lck^ complexes serve as the initiators of the protein tyrosine phosphorylation cascade in T-cells. As we stated: “an association between the T4 (CD4) receptor and the PTK within the cell would introduce a specific pathway by which T-cells become activated. The T4 (CD4)-associated kinase could act to phosphorylate various intracellular candidates. An obvious and important candidate would be the subunits of the T3-Ti antigen receptor complex.” We envisioned this to occur during antigen-presentation by dendritic cells due to CD4 or CD8 and the TCR coordinate binding to MHC antigens. This event would bring p56^lck^ into close physical proximity where trans-phosphorylation could occur. In agreement with this model, using substrate phosphorylation assays, we showed that CD4 and CD8-p56^lck^ could trans-phosphorylate the TCR-ζ and the CD3γ, δ, ε chains on tyrosine residues (Barber et al., 1989). We even observed that anti-CD4 co-precipitated TCR-ζ and all CD3 chains phosphorylated on tyrosine residues (Burgess et al., 1991). Antibody cross-linking of CD4 was also found result in the zeta chain phosphorylation on tyrosine residues (Veillette et al., 1989). Together, these observations fit nicely with the finding of tyrosine phosphorylation of the human TCRζ chain in hybridomas (Weissman et al., 1988) and in T-cells from patients with lymphoproliferative disorders (Samelson et al., 1986a), but additionally, implicated the CD3 subunits as targets of p56^lck^. Subsequent imaging studies underscored the importance of the spatial distribution of TCR and p56^lck^ in the initiation of T-cell signaling (Purbhoo et al., 2010; Rossy et al., 2012). Antigen-engaged TCRs may scan for co-receptors coupled to p56^lck^ as a rate-limiting step in T-cell activation (Stepanek et al., 2014).

Subsequent work showed that p56^lck^ binding to CD4 also masks a key dileucine motif required for clathrin-mediated endocytosis of CD4 is masked by p56^lck^ (Kim et al., 2003). Although not well-publicized, this observation suggests a second function for p56^lck^ binding to CD4 in increasing the lifespan of CD4 on the surface of T-cells for the generation of activation signals. Following T cell activation, p56^lck^ dissociates from CD4 allowing the coreceptor to be internalized (Pelchen-Matthews et al., 1992, 1993).

Shortly after the 1988 papers, Michael Reth identified a consensus sequence (D/E)xxYxx(I/L)x6–8Yxx(I/L) in the TCR associated chains (Reth, 1989), motifs that eventually became known as the immuno-receptor tyrosine-based activation motifs (ITAMs) (Cambier, 1995). TCR-CD3ζ homodimer possesses six ITAMs while ITAMs existed in the CD3 subunits, each carrying one ITAM. The presence of the ITAMs in both the CD3 and zeta subunits fit nicely into our observations that the various chains were all phosphorylated by p56^lck^. ITAMs were found also in the CD79-alpha and -beta chains of the B cell receptor complex, certain Fc receptors and other receptors (Zettlmeissl et al., 1990).

A major question that persists today is why are there so many ITAMs within a single receptor complex as targeted by p56^lck^? Is it a case of evolutionary redundancy, dosage compensation or do different ITAM send unique signals? Several groups heroically attempted to define a precise order of phosphorylation of the CD3ζ tyrosine residues (Kersh et al., 1998; Housden et al., 2003). ^1^H-NMR studies of recombinant zeta chain have shown p56^lck^ sequential phosphorylation of the TCRζ N-terminal tyrosine (N1) first followed by 3N >3C >2N >1C >2C (Housden et al., 2003). The efficacy of ITAM phosphorylation also depends on the accessibility of the cytoplasmic tails. The CD3 subunits and zeta chains lie attached at the inner layer of the plasma membrane due to electrostatic interactions with phosphoserine (PS) (Shi et al., 2013). This feature protects ITAMs from spontaneous phosphorylation (Xu et al., 2008; Ma et al., 2017) and accessibility to p56^lck^ (Gil et al., 2002). In this model, increased intracellular calcium and its binding to negatively charged PS may free the CD3-zeta subunits cytoplasmic tails for CD4 and CD8-p56^lck^ access and phosphorylation. It remains uncertain whether the sequential phosphorylation by p56^lck^ of ITAMs has a physiological role in regulating T-cell immunity.

Nevertheless, increasing phospho-ITAMs has been reported to correlate with distinct T cell responses, such as activation, anergy, or apoptosis (Sloan-Lancaster et al., 1994; Madrenas et al., 1995; Combadiere et al., 1996; Kersh et al., 1998). Others have documented a linear correlation between the number of wild-type CD3 ITAMs and T cell proliferation, but not in terms of cytokine production (Holst et al., 2008). A low number of TCR-CD3 ITAMs suffices to support cytokine secretion (Guy et al., 2013). However, despite this effort, a seminal paper from the from the lab of Marie and Bernard Malissen showed that the crippling of zeta ITAMs did not impair T cell receptor signaling and only marginally affected T-cell responses to antigen *in vivo* (Ardouin et al., 1999). It, therefore, appeared that the ITAMs in the remaining CD3 subunits sufficed to generate signals needed for *in vivo* responses to antigen. It may, therefore, be possible that the multiplicity of ITAMs regulates proliferation to antigens of low affinity or abundance. From another direction, an interesting study from the lab of Dario Vignali documented a role for multiple ITAMs in thymic selection which discriminates self-antigen on the basis of affinity. Mice with fewer than seven wild type TCR ITAMs developed a lethal, multiorgan autoimmune disease due to defective central tolerance (Holst et al., 2008).

Whether access to glycosphingolipid enriched microdomains (GEMs) or rafts is needed is an open question (Pizzo and Viola, 2003). Rafts are enriched with SFKs (Bunnell et al., 2002) where in the case of p56^lck^, lipidation targets the kinase to lipid rafts (Rodgers et al., 1994). TCR and CD4/CD8 also move into rafts during the TCR ligation process. The activating complexes in rafts facilitates p56^lck^ phosphorylation CD3 phosphorylation and activation (Arcaro et al., 2001), although others have reported that the kinase in these domains has low activity due to the action of the CBP/PAG/CSK inhibitory complex (Kabouridis, 2006). On the other hand, expression of a mutant construct of p56^lck^ with a transmembrane domain that is excluded from rafts was unable to phosphorylate the TCR (Kabouridis et al., 1997). Due to the fact that the TCR is not raft-associated in resting T cells, these microdomains are likely to play greater roles in maintaining rather than initiating TCR signaling. It is worth noting that cholesterol-rich rafts are also modulated by co-receptors CD28 which promote and CTLA-4 which disassemble the domains (Martin et al., 2001).

## Regulation of the CD4/CD8-p56^lck^ Complex

While the regulation of signaling via receptors with intrinsic domains such as the PDGF-R involves dimerization and is well-understood, the mechanism underlying the function of the CD4 and CD8-p56^lck^ complexes is complex and still unresolved. Certain models involve cross-regulation by transmembrane and intracellular phosphatases and kinases, while other models involve the simple dimerization independent of phospho-regulation (Cooper and Qian, 2008). The crosslinking of CD4 with antibody can increase p56^lck^ activity; however, it is unclear that CD4 actually dimerizes during antigen-presentation (Veillette et al., 1989). Similarly, while CD4 and CD8-p56^lck^ complexes aggregate in microdomains and at the immunological synapse (IS), it is unclear whether this is mimics the close proximity of receptors induced by antibody crosslinking. Further, microdomains include the aggregation of numerous other immunoglobulin family members that could complete, or sterically interfere with potential CD4 and CD8 inter-molecular receptor interactions. Although enhanced p56^lck^ activities has been seen in membranes expressing CD4 or CD8 (Liaunardy-Jopeace et al., 2017), the lab of Oreste Acuto found that some 40 per cent of total p56^lck^ in naive T cells is constitutively active (Nika et al., 2010). Intriguingly, TCR and coreceptor engagement did not change the levels of activate p56^lck^ even though TCR ζ phosphorylation was observed (Nika et al., 2010). Overall, it remains an open question whether an increase in p56^lck^ catalytic activity is needed for the function of the CD4 and CD8-p56^lck^ complexes, or whether the simple localization of constitutively active p56^lck^ next to key substrates such as the ITAMs of TCRζ and CD3 chains is sufficient to initiate the activation cascade, as we originally proposed (Rudd et al., 1988; Barber et al., 1989; Rudd, 1990).

p56^lck^ has a classic structure involving an N-terminal src homology domain (SH4) that is myristoylated at Gly2 and palmitoylated at Cys3 and Cys5 (Kabouridis et al., 1997). The latter modification is needed for membrane binding and p56^lck^ diffusion to the IS (Yurchak and Sefton, 1995). Interestingly, all SFKs have palmitoylate linkages except Src and Blk. This region is followed by poorly conserved unique region, an SH3 domain that binds to proline-rich residues, an SH2 domain that binds to phospho-tyrosine motifs, a linker region, the SH1 kinase domain followed by a C-terminal negative regulatory region (Figure 1B). Within the kinase, there is an autophosphorylation site within the activation loop of the catalytic domain at residue Y-416 for pp60^src^ and Y-394 for p56^lck^. At the C-terminus, there is a key negative regulatory residue at Y-527 for pp60^src^ and Y-505 for p56^lck^ (Martin, 2001). p56^lck^ is distinguished by an N-terminal CxxC motif in the SH4 domain that coordinates Zn^2+^ binding in a zinc clasp with CD4 and CD8 (Huse et al., 1998; Lin et al., 1998; Kim et al., 2003). Our initial comparison of the cytoplasmic tails of CD4 and CD8 identified homologous motifs, Thr-Cys-Gln-Cys-Pro-His in CD4 and Val-Cys-Lys-Cys-Pro-Arg in CD8 for p56^lck^ binding (Barber et al., 1989). It was evident that the β chain of CD8 did not have the motif (Barber et al., 1989). A more refined analysis identified conserved cysteines within a CxCP motif of CD4 and CD8α (Rudd et al., 1989; Shaw et al., 1990; Turner et al., 1990).

**Figure 2:**
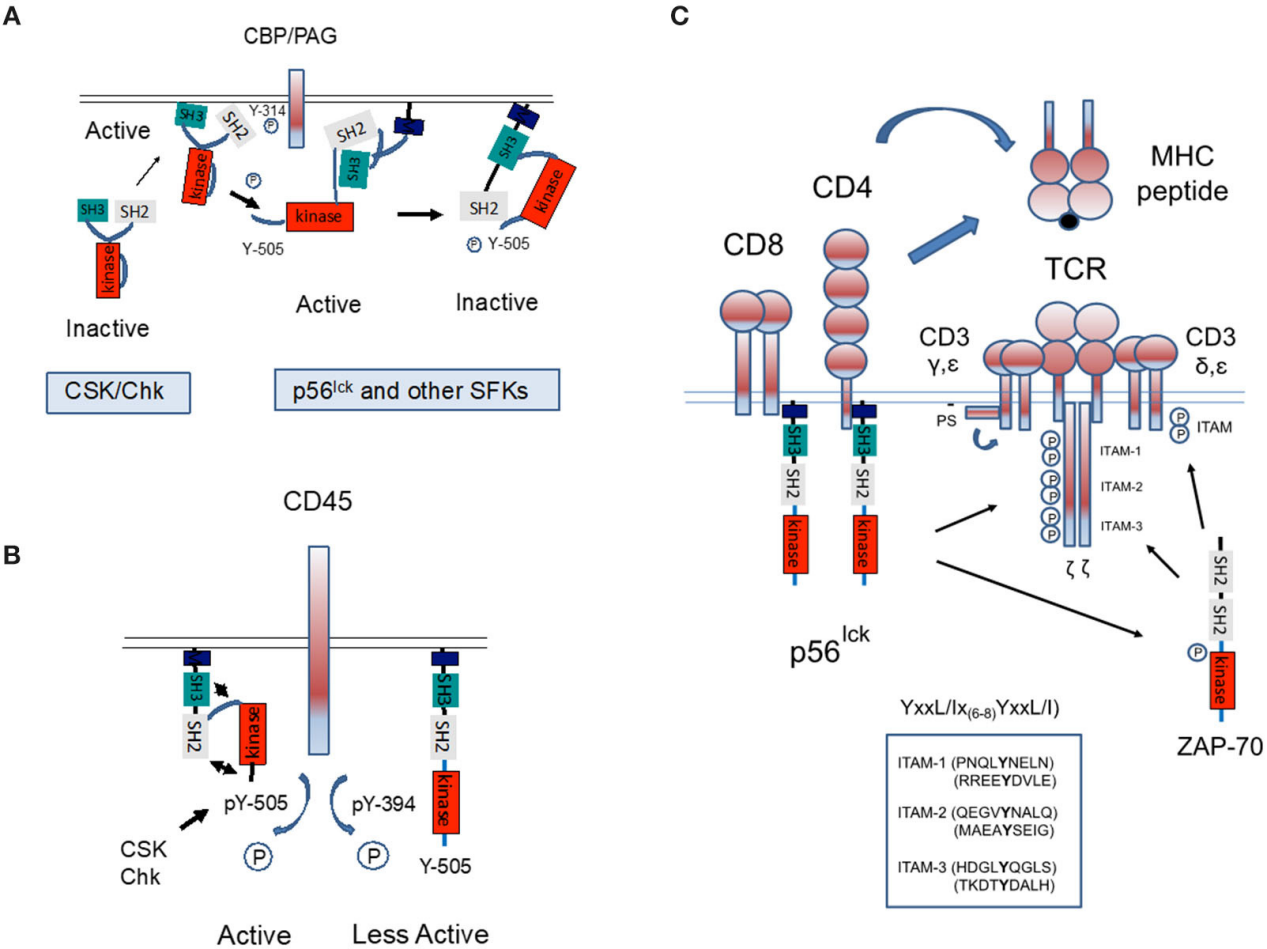
Regulation of p56^lck^ and phosphorylation of the TCR complex. **(A)** Regulation of p56^lck^ kinase activity. The SH2 domain of p56^lck^ binds to the C-terminal inhibitory Y-505, an interaction aided by SH3 domain binding to proline residues. These interactions hold the structure in a closed inactive conformation. Phosphorylation of the C-terminal Y-505 is inhibitory, while the dephosphorylation at Y-505 unfolds the kinase, unleashing its full catalytic activity accompanied by auto-phosphorylation of Y-384 within the catalytic domain. In this context, C-terminal protein kinase and related CSK-homologous kinase (Chk) bind to the anchoring protein CBP/PAG and inactivates p56^lck^ by phosphorylation on Y-505. **(B)** The transmembrane protein phosphatase CD45 counterbalances the effect of CSK by preferentially dephosphorylating the inhibitory Y-505 tyrosine. However, CD45 can also dephosphorylates Y-394 to dampen kinase activity. The relative effects on Y-505 and Y-394 may be temporally regulated. **(C)** Model whereby CD4 and CD8-p56^lck^ phosphorylate ITAMs on the TCRζ and CD3γ, δ, ε chains. During antigen-presentation by antigen-presenting cells (i.e., dendritic cells), coordinate binding of CD4/CD8 and the TCR to MHC antigens would bring p56^lck^ into proximity where trans-phosphorylation would occur. p56^lck^ also phosphorylates and activates ZAP-70.

In an inactive conformation, p56^lck^ is folded in upon itself as mediated by intra-molecular binding of the SH2 domain to the C-terminal inhibitory Y-505, an interaction aided by SH3 domain binding to the linker region (Xu et al., 1995). These interactions hold the structure in a closed inactive conformation (Figure 2A). Dephosphorylation at Y-505 is sufficient to unfold the kinase, holding the kinase in a primed conformation which requires autophosphorylation at Y-394 for full kinase activity.

C-terminal phosphorylation is regulated by inhibitory kinases and a stimulatory phosphatase. The kinases, C-terminal Src kinase (CSK) and the related CSK-homologous kinase (Chk) phosphorylate the C-terminal tyrosine, thereby inhibiting p56^lck^ (Bergman et al., 1992; Figure 2A). Key to CSK function is the transmembrane adaptor termed CSK-binding protein (CBP/PAG). When phosphorylated, CBP/PAG recruits CSK to the membrane for its activation and access to SFKs. The de-phosphorylation of PAG causes a loss of CSK from the vicinity of the TCR (Horejsi, 2004). CSK lacks N-terminal acylation sites, an autophosphorylation site and C-terminal regulatory sites found in p56^lck^. The C-terminal tyrosine of SFKs may be the only substrate of CSK (Brown and Cooper, 1996). Unlike SFKs, the SH2 and SH3 binding pockets of CSK appear oriented outwards (Ogawa et al., 2002). They inhibit SFKs due to phosphorylation but also possibly by direct binding (Chong et al., 2005). CSK itself is phosphorylated and positively regulated by cAMP-dependent protein kinase (PKA) (Vang et al., 2001). In one model, CSK is activated by CBP/PAG in glycosphingolipid enriched microdomains (GEMs) (or rafts). Overall, the CBP/PAG-CSK complex is likely to maintain T-cells in a quiescent state until there is a requirement for activation signals.

Another key regulator of p56^lck^ is the transmembrane phosphatase (PTPase) CD45 (Mustelin et al., 2002). First identified by the lab of Alan Williams in Oxford, and termed leucocyte common antigen (L-CA), it is an immune specific and unusually abundant protein on T-cells (Barclay et al., 1988; Figure 2B). It is highly conserved, comprising as much as 10% of protein on the surface of cells (Barclay et al., 1988). Structurally, it contains an extended extracellular domain, and two tandem intracytoplasmic catalytic PTPase domains (Tonks et al., 1990). We and others showed that CD45 is also processed into different isoforms (Rudd et al., 1987; Takeuchi et al., 1989), which define different subsets of T-cells (Wallace and Beverley, 1990). Naive T lymphocytes are positive for CD45RA with only the A protein region of the differentially spliced protein. By contrast, activated and memory T lymphocytes express CD45RO, the shortest isoform lacking all three of the A, B, and C regions.

Despite its clear importance, the nature of CD45 function and the relevance of the different isoforms continues to confound investigators since it appears to act as a positive and negative regulator (Charbonneau et al., 1989; Mustelin et al., 1989; McNeill et al., 2007; Courtney et al., 2019). Early studies showed that CD45 dephosphorylates Y-505 and activates p56^lck^ (Mustelin et al., 1989), while the Ashwell lab showed that it also acts on the autophosphorylation site Y394 to inhibit full p56^lck^ activity (Ashwell and D'Oro, 1999; Figure 2B). As evidence in support of a positive function, certain CD45-negative T cells fail to respond to TCR stimulation and increased CD45 expression correlates with increased sensitivity to TCR ligation (Koretzky et al., 1990; Cahir McFarland et al., 1993). However, others have found that with the inhibition of CSK, CD45 suppresses ζ-chain phosphorylation and alters the pool of active p56^lck^ (Courtney et al., 2019). The kinetic-segregation model of TCR triggering excludes CD45 with its large ectodomain from ligated TCRs (Shaw and Dustin, 1997; Davis et al., 2003). CD45 may have different functions which depend on expression levels, adjacent regulatory molecules and the temporal stage of T-cell activation. In one model, the transient appearance of CD45 in rafts lead to p56lck dephosphorylation and activation. The field is further complicated by its dephosphorylation JAK (Janus kinase) kinases and its negative regulation of cytokine receptor signaling as well as in the negative regulation of other cells such as monocytic and erythroid differentiation (Irie-Sasaki et al., 2001). Further, CD45 seems to have different effects on different SFKs (Roach et al., 1997). Added to the mix, the cytoplasmic phosphatase SHP-1 also dephosphorylates at Y-394 to limit T-cell activation (Chiang and Sefton, 2001; Nagaishi et al., 2006).

## CD4/CD8-lck Initiate the T-cell Tyrosine Phosphorylation Cascade

Aside from ITAMs, a second major substrate of p56^lck^ is the protein-tyrosine kinase, zeta-chain associated protein kinase 70 (ZAP-70). We originally found that CD4-lck precipitated two other bands that were labeled on tyrosine residues at 38–40 Kd and 70–80 Kd in *in vitro* kinase assays (Rudd et al., 1988). Our initial precipitates showed that anti-SYK (spleen tyrosine kinase) was able to precipitate the 75 Kd protein; however, due to the limited quantity of the antisera available at the time, the results were considered unreliable. SYK had been described in B-cells as a novel protein tyrosine kinase with two tandem SH2 domains separated by a long linker (linker B) from a C-terminal kinase domain. Instead, a major seminal advance came from the lab of Art Weiss with the cloning of the 70 Kd band corresponding to Zeta-chain-associated protein kinase 70 (ZAP-70) (Chan et al., 1992). Similar to p56^lck^, ZAP-70 is primarily expressed in T- and natural killer cells; however, it is structurally homologous to SYK with two SH2 domains that bind to two tandem tyrosines in each ITAM. p56^lck^ phosphorylates both ITAMs needed for ZAP-70 recruitment and sites within ZAP-70 needed for its activation (Iwashima et al., 1994; Chan et al., 1995; Figure 2C).

Importantly, in the context of the tyrosine phosphorylation cascade, the range of substrates of p56^lck^ and ZAP-70 are profoundly different. As will be reviewed, while p56^lck^ and related SFKs phosphorylate a broad spectrum of substrates needed for the phosphorylation cascade, ZAP-70 phosphorylates only a few known candidates to date, such as LAT (linker of activated T cells) and SLP-76 (SH2-domain-containing leukocyte protein of 76 kD). This fits with the notion that the p56^lck^ is responsible for the main wave of tyrosine phosphorylation cascade of numerous substrates that includes ZAP-70 with a more specialized function in phosphorylating a limited additional number of key substrates needed for specific functions such as calcium mobilization.

**Figure 3:**
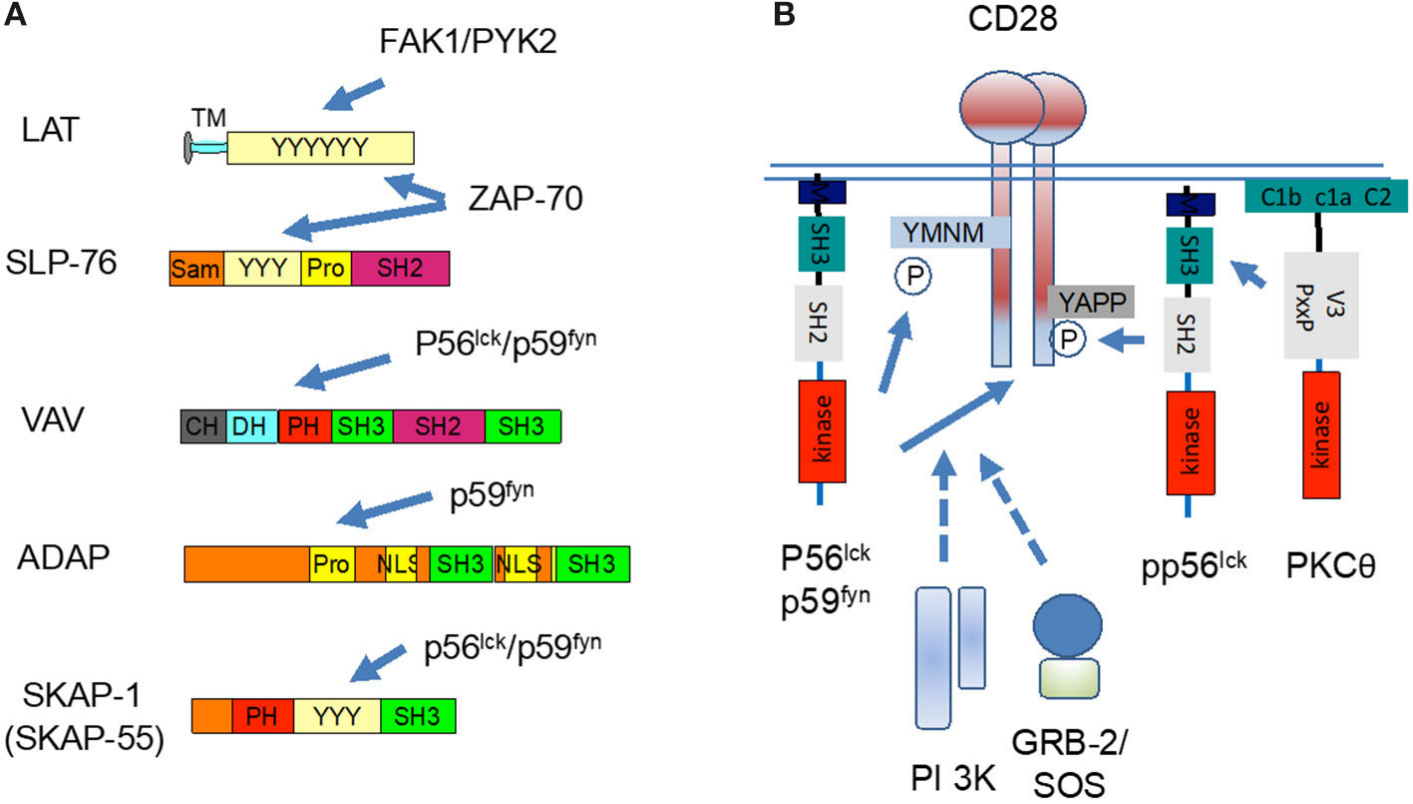
p56^lck^ regulates the function of immune adaptors and CD28 co-stimulation. **(A)** p56^lck^ and related p59^fyn^ phosphorylate immune specific adaptors or molecular scaffolds. These include LAT, SLP-76, VAV, ADAP, and SKAP1. The C-terminal SH2 domain SLP-76 binds to the ADAP, while ADAP binds to SKAP1. **(B)** p56^lck^ and p59^fyn^ phosphorylate the cytoplasmic tail of CD28 YxxM site for the binding of PI 3K and GRB-2/ SOS. CD28 also have a more distal YAPP site which when phosphorylated, binds to the SH2 domain of p56^lck^. The V3 domain of PKC-θ, in turn, binds to CD28 via binding to Lck. CD28 and PKCθ co-localize and act as markers for the c-SMAC.

Part of the overall cascade includes immune cell-specific adaptors, proteins that lack enzymatic activities, and instead are made up of domains or sites that mediate complex formation (Rudd, 1999). They are considered types of molecular switches which integrate proximal signaling with downstream events. Key examples include LAT, SLP-76, ADAP (adhesion and degranulation-promoting adapter protein, also known as Fyn-binding protein [Fyb] or SLP-76 associated protein of 130 kD [SLAP-130]) and SKAP1 (or SKAP-55, Src kinase-associated phosphoprotein of 55 kDa; Figure 3A).

**Figure 4:**
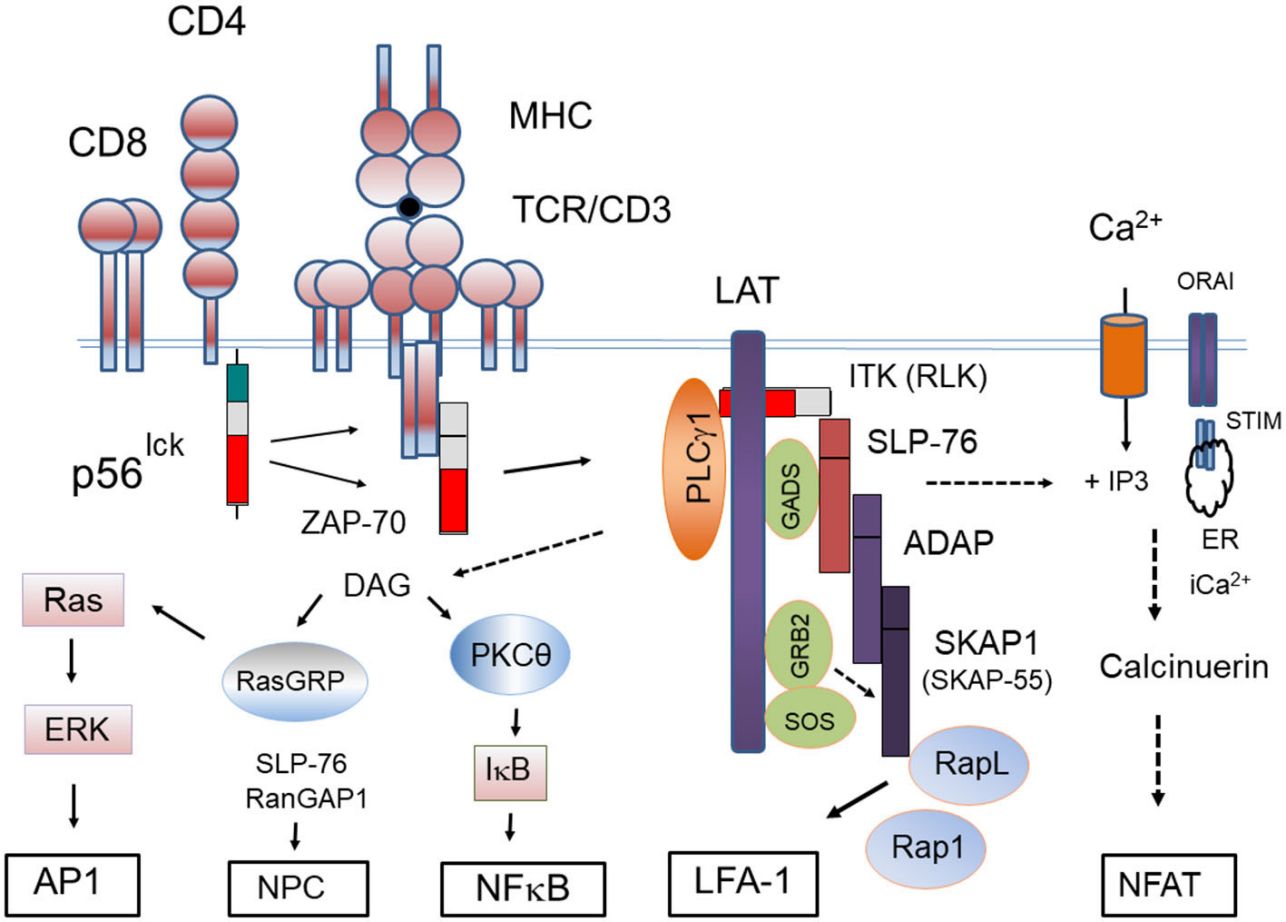
Proximal signaling complexes and downstream responses initiated by the CD4/CD8-p56^lck^ complexes. Model outlining CD4/CD8-p56^lck^ initiation of the protein-tyrosine activation cascade. CD4/CD8-p56^lck^ phosphorylation of TCR ITAMs leads to the recruitment and activation of ZAP-70 followed by its phosphorylation and formation of the LAT signalosome. pLAT recruits several SH2-domain-containing proteins, including phospholipase Cγ-1 (PLCγ1) growth factor receptor-bound protein 2 (GRB2) and GRB2-related adaptor protein (GADS). Through its constitutive association with GADS, SLP-76 constitutively associates with LAT. Associated IL-2-inducible T-cell kinase (ITK) and resting lymphocyte kinase (RLK) phospho-activate PLCγ1 resulting in the hydrolysis of phosphatidylinositol 4,5-bisphosphate to inositol 3,4,5-triphosphate (IP3) and diacylglycerol (DAG). IP_3_ production leads to increases of intracellular free Ca^2+^ concentration, whereas DAG can activate both protein kinase C- (PKC-θ) and RAS guanyl nucleotide-releasing protein (RASGRP). IP_3_ generated from PIP_2_ binds to the Ca^2+−^permeable ion channel receptors (IP3R) in the endoplasmic reticulum (ER) releasing Ca^2+^ from within ER stores to the cytoplasm. The ER senses intracellular Ca^2+^ levels through stromal interaction molecule (STIM). Depletion of intracellular Ca^2+^ triggers an Ca^2+^influx from Orai1 type plasma membrane calcium-release activated calcium (CRAC) channel. Increased intracellular Ca^2+^ activates a protein phosphatase, calcineurin, that dephosphorylates the nuclear factor of activated T cells (NFAT) for its nuclear translocation. pLAT also recruits the SH2 domain of GRB2 and GRB2-associated RAS guanosine nucleotide-exchange factor (GEF), son-of-sevenless (SOS) to activate p21^RAS^. Tyrosine-phosphorylated SLP-76 also associates with the immune cell adaptors ADAP and SKAP1. SKAP1 controls the formation of the Rap1-RapL complex needed for LFA-1 activation. SLP-76 also interacts with RanGAP1 in the nuclear complex for the increased transport of transcription factors NFAT and NFkb into the nucleus.

LAT, as first identified by the lab of Larry Samelson at the NIH, is a transmembrane adaptor with multiple tyrosine residues that binding SH2 domain carrying mediators, phospholipase Cγ1 (PLCγ1) (Y-132) and the small adaptors, Growth factor receptor-bound protein 2 (GRB-2) (Y-171, 191, and 226), and GRB2-related adapter protein 2 (GADs) (Y-171 and 191) (Zhang et al., 1998, 2000). ZAP-70 phosphorylates LAT at all sites needed for recruitment (Bunnell et al., 2000; Zhang et al., 2000). Mutation of individual sites does not prevent GRB2 binding, while the double mutation of Y-171 and Y-191 abolishes GADs binding. Overall, there is cooperativity in the binding of different molecules, including PLCγ1 (Cho et al., 2004). Significantly, LAT deficient Jurkat cells show normal phosphorylation of the TCR complex and ZAP-70 activation, but are defective downstream in the activation of PLCγ1, extracellular-signal-regulated kinases (ERKs) as well as interleukin 2 transcription (Finco et al., 1998). Further, *Lat*^−/−^ mice showed defects in thymic differentiation with a block at the double negative 3 stage (Samelson et al., 1999). The GADs SH3 domain binds to SLP-76 with an unusually high avidity (Berry et al., 2002), bringing the complex with SLP-76 into the LAT signalosome (Figure 4).

Recently, we uncovered an unexpected connection between integrin signaling and LAT phosphorylation (Raab et al., 2017). LFA-1 ligation and crosslinking activated the protein-tyrosine kinases FAK1 and PYK-2 to phosphorylate LAT at a single site at Y-171. The specificity and fidelity of phosphorylation was remarkable as it was seen in *in vitro* and *in vivo* assays. Further, the specificity of FAK1 and PYK-2 contrasts with ZAP-70 phosphorylation of the three LAT sites. It appeared to compete with the action of ZAP-70 acting mostly in the actin-rich periphery of the contact area of T-cells and recruited GRB-2-SKAP1 in the control of adhesion (Raab et al., 2017).

SLP-76, first identified by Jackman et al. (1995), has an N-terminal sterile-α motif (SAM) and a carboxy-terminal SH2 domain that binds to ADAP (da Silva et al., 1997a; Musci et al., 1997) and the hematopoietic progenitor kinase-1 (HPK-1) (Di Bartolo et al., 2007). SLP-76 is needed for phospholipase Cγ1 (PLCγ1) activation, calcium mobilization and thymic differentiation (Jordan et al., 2003). We and others showed that ZAP-70 also phosphorylates SLP-76 at two specific sites (Y113 and Y128) (Bubeck Wardenburg et al., 1996; Raab et al., 1997). p59^fyn^ was also found to phosphorylate the adaptor with unclear consequences (Raab et al., 1997). Lastly, in an unexpected manner, following TCR ligation, we have found that SLP-76 interacts with RanGAP1 of the nuclear pore complex where it promotes NFAT and Nfkb entry into the nucleus (Liu et al., 2015).

A key hallmark consequence of LAT phosphorylation is the phospho-activation phospholipase Cγ-1 (PLCγ1) (Samelson et al., 1995). PLCγ1 phosphorylation is regulated by protein tyrosine kinase-mediated phosphorylation induced by TCR ligation (Mustelin et al., 1990), however, the molecular steps involved had been unclear for decades. Early studies had shown that the loss of SLP-76 was associated with a selective loss of PLCγ1 and calcium mobilization in T-cells (Yablonski et al., 1998). It was then shown that LAT docking of PLCγ1 and SLP-76 facilitates the binding of another kinase, IL-2-inducible T-cell kinase (ITK), which phosphorylates PLCγ1 for activation (Berg et al., 2005). ITK-related resting lymphocyte kinase (RLK) also contributes (Sommers et al., 1999; Schneider et al., 2000). In fact, the deletion of both ITK and RLK eliminates PLCγ1 activity accompanied by defects in calcium flux following TCR engagement (Schaeffer et al., 1999). These discoveries unraveled a longstanding puzzle in T-cell signaling. Activation of PLCγ1 results in the hydrolysis of phosphatidylinositol 4,5-bisphosphate to diacylglycerol (DAG) and inositol 3,4,5-triphosphate (IP3). DAG activates protein kinase C (PKC-θ) and RAS guanyl nucleotide-releasing protein (RASGRP) for activation of the p21^ras^ and ERK pathways (Figure 4). IP_3_ binds to calcium permeable ion channel receptors (IP3R) in the endoplasmic reticulum (ER) which releases the ion into the cytoplasm. The ER also detects intracellular Ca^2+^ through stromal interaction molecule (STIM). Intracellular Ca^2+^ depletion triggers an influx from outside the cells as mediated by calcium-release activated calcium (CRAC) channel. Increased intracellular Ca^2+^ activates the phosphatase, calcineurin, which in turn dephosphorylates the nuclear factor of activated T cells (NFAT) for entry into the nucleus (Jain et al., 1992). Overall, CD4/CD8-p56^lck^ phosphorylation of the TCR/CD3 subunits sets in motion a cascade where ZAP-70 is recruited leading to the phosphorylation of LAT for PLCγ activation, the mobilization of calcium and the translocation of NFAT into the nucleus of T-cells.

Further, calcium may bind and neutralize PS facilitating the release of the cytoplasmic CD3 and zeta chains from the inner leaflet of the plasma membrane (Shi et al., 2013). The association normally protects ITAMs from spontaneous phosphorylation (Xu et al., 2008; Ma et al., 2017). However, with activation, antigen-receptor ligation would render ITAMs more accessible to p56^lck^ (Gil et al., 2002).

We and others have shown that ZAP-70 phosphorylates SLP-76 at residues Y-113 and Y-128 for binding to the guanine nucleotide exchange factor (GEF), VAV-1 and another adaptor NCK (Bubeck Wardenburg et al., 1996; Raab et al., 1997, 2001; Michel et al., 1998; Rudd and Raab, 2003). VAV-1 is a member of the Dbl GEF family with activity against for the Rho family of GTP binding proteins. GEFs activate by catalyzing the exchange of guanosine diphosphate (GDP) for guanosine triphosphate (GTP). Effectors of Vav1 include RhoA, Rac1, and Cdc42 which play central roles in cytoskeleton organization, cell polarity and movement. p59^fyn^, p56^lck^, and ZAP-70 phospho-activate VAV-1 activity (Michel et al., 1998). Vav cooperates with CD28 to induce NF-kB activation via a pathway involving Rac-1 and mitogen-activated kinase (Marinari et al., 2002). The activation of protein kinase B (PKB/AKT) and glycogen synthase kinase-3 (GSK-3) operates independently of VAV-1 (Wood et al., 2006).

Further along the cascade, my lab and others showed that SLP-76 binds to the immune cell adaptor ADAP which, in turn, binds to another immune cell adaptor, SKAP1 (or SKAP55) (da Silva et al., 1997a; Wang et al., 2004; Kliche et al., 2006). SKAP1 had a unique N terminus, a PH domain and a C terminal SH3 domain (Marie-Cardine et al., 1997). The C-terminal SH2 domain SLP-76 binds to the ADAP (da Silva et al., 1997a; Musci et al., 1997; Liu et al., 1998; Veale et al., 1999), while ADAP binds to SKAP1 (Marie-Cardine et al., 1997; Liu et al., 1998). SKAP1 SH3 domain binds to proline residues in ADAP, while the ADAP-SH3-like domain binds to SKAP1 (Kang et al., 2000; Kliche et al., 2006). SKAP1 is an effector in the pathway such that the Rap1-RapL complex fails to form in *skap1*^−/−^ T cells, which correlates with reduced LFA-1 binding to ICAM-1 and T-cell adhesion to dendritic cells (DCs) (Wang et al., 2003; Raab et al., 2010, 2011, 2018). Rap1 also interacts with Rap1-GTP-interacting adaptor molecule (RIAM) which controls recruitment of the cytoskeletal protein and integrin-binding protein, talin, to the membrane (Lafuente and Boussiotis, 2006). In this manner, SKAP1 and RIAM couples the TCR to the activation of the integrin, LFA-1 which is needed to promote the binding of T-cells to antigen-presenting cells (Wang et al., 2003, 2004, 2009; Menasche et al., 2007).

## Other Substrates

p56^lck^ and ZAP-70 differ in their phosphorylation specificities. p56^lck^ phosphorylates a wide range of downstream targets that regulates functions as diverse as cell movement, cell cycle, metabolism, cell to cell interactions, morphology, protein synthesis, and gene expression. The main problem in identifying SFK substrates has been the reliance on the use of oncogenic forms of *src* kinases. These versions of the kinases are likely unreliable since their constitutive kinase activities allow for the phosphorylation of secondary targets not engaged by the non-oncogenic forms of the kinase. To this end, elegant add-back experiments have been conducted with c-src (Amanchy et al., 2009; Ferrando et al., 2012). With the qualifier that *c-src* is not palmitoylated, these studies are likely to give an idea of the range of substrates engaged by p56^lck^ since the kinase domains of pp60^c−src^ and p56^lck^ are highly conserved. As seen in Table 1, c-src substrates include epidermal growth factor receptor substrate 15 (Eps15) with a role in the assembly of clathrin-coated pits, Tripartite motif protein 28 (TRIM28) involved in transcriptional regulation, cellular differentiation and proliferation, DNA damage repair and apoptosis, Xanthine dehydrogenase (XDH) involved in the oxidative metabolism of purines, Seryl-aminoacyl-tRNA synthetase 1, Guanine monophosphate synthetase eEF 2, and Threonyl-tRNA synthetase involved in protein translation, the protease Calpain 2 and Unc-84 homolog, a nuclear envelope protein. Others include Heat shock protein 9A and Stress-induced phosphoprotein 1 and Heat shock protein 1 (chaperonin) (Amanchy et al., 2009; see Table 1). Further, others include cytidine 5-triphosphate (CTP) synthase phosphorylation on multiple sites (Huang and Graves, 2003), pyruvate kinase 3 (type M2) (Eigenbrodt et al., 1992), and valosin containing protein (VCP) which involved in the proteolytic degradation of misfolded proteins (Song et al., 2008). Further, there are phospho-targets involved in adhesion such as Talin, Tensin1-2, FAK, and p130Cas and others involved in actin remodeling as well as others, such as filamin B, ABLIM1, and PARD3 that regulate cell polarity. C3G is a guanine nucleotide exchange factor for the small Ras-related G-proteins Rap1, Rap2, and R-Ras (Ferrando et al., 2012; Sasi Kumar et al., 2015). Rap1 is a small G-protein of the Ras family that antagonizes Ras in some cells (but not T-cells) (Sebzda et al., 2002), and has been implicated in SKAP1 activation of integrin adhesion in T-cells (Raab et al., 2010). CasL, DOK1, and GAB1 are also putative targets. Overall, SFKs intersect in the regulation of FAK, integrin, PAK and PTEN signaling, amongst others (Ferrando et al., 2012). Although targets will vary depending on the localization of each kinase, this approach provides a hint of the array of substrates in the CD4 and CD8-p56^lck^ initiated phosphorylation cascade, linked to functions as diverse as translation, gene expression and metabolism in T-cells.

**Table 1:** p56^lck^ predicted substrates.

**Adhesion**	**Kinases**	**Cellular functions**	**Adaptors**	**Functions**
Talin KIRREL1 PCDH19 Tensin1-2 MAGI1 PXN FAK p130Cas	Hck ERK1/2 ICK PIK3R2 ARG	Eps15 Tripartite motif protein 28 UAP1 like-1 Xanthine dehydrogenase Seryl-aminoacyl-tRNA synthetase 1 Calpain 2 Unc-84 homolog Heat shock protein 9A Threonyl-tRNA synthetase Stress-induced phosphoprotein 1 Guanine monophosphate synthetase eEF 2 Calnexin ATP citrate lyase Heat shock protein 1 Cytidine 5-triphosphate (CTP) synthase Pyruvate kinase 3 Valosin	GAB1 Cas-L PZR DOK1 ABI1/2 IRS1 ANKS1 CRKL ZO-1 RaspL1 HGS LPP SHC1 Shc1 LAT SLP-76	Cell movement Cell cycle Metabolism Cell to cell interactions Cell morphology Protein synthesis Gene expression
	**PTPase**			
**Actin re-modeling and polarity**	PTPRA			
Filamin B ABLIM1 PARD3 PARD3B	**Others** TTYH2 TMEM106B ZDHHC8 P53 ST5 Tenacin PGAM1 RPL15			
**GEF/GAP**				**Other kinases**
GIT1/2 ARHGAP32 C3G				FAK signaling Integrin signaling Ephrin signaling ERL signaling PAK signaling PTEN signaling

*Adapted from Amanchy et al. (2009) and Ferrando et al. (2012)*.

### CD8α/α vs. CD8α/β

As mentioned, the CD8 coreceptor is expressed as an α/α homodimer and an α/β heterodimer. It is the α chain of the CD8 complex that binds to major histocompatibility complex leukocyte antigens (Gao et al., 1997) and non-classical MHC antigens such as the human histocompatibility leukocyte antigen G found on trophoblast cells (Sanders et al., 1991). With two chains to bind to p56^lck^, CD8α/α has the potential to be hyper-stimulatory; however, paradoxically, we and others have found less kinase activity associated with this form of the co-receptor. The molecular basis for this is not known but might involve conformational or trans-phosphorylation issues. Trans-phosphorylation occurs between separate receptors, but within the same covalently linked receptor complex, autophosphorylation might become disordered in some manner.

Similar to other activation antigens such as CTLA-4, CD8α expression is induced by TCR ligation proportional to the strength of signal. In the case of CD8α/β, it is expressed at higher levels in T-cell lines sensitive to TCR engagement (Cawthon et al., 2001) and down-regulated in response to an altered peptide ligand (Barnden et al., 1997). Further, CD8β couples the TCR/CD3 complex to rafts (Arcaro et al., 2001). By contrast, the expression of CD8α/α decreases the functional avidity of TCRs and reduces activation (van Oers et al., 1993). Furthermore, unlike in the case of activation-induced co-internationalization of TCR and the CD8α/β complex, CD8α/α is excluded from lipid rafts (Pang et al., 2007). In one model, CD8α/α sequesters p56^lck^ from rafts leading to a reduction in the TCR phosphorylation. Collectively, this has led to the hypothesis that CD8α/α may act an inhibitory receptor, possibly antagonizing the function of CD8α/β in promoting activation (Cheroutre and Lambolez, 2008). The antagonism may promote the differentiation of activated lymphocytes into memory CD8 T cells (Madakamutil et al., 2004).

## p56^lck^ and CD28 Mediated Co-stimulation

Although initially discovered in the context of TCR signaling, subsequent work implicated the p56^lck^ and related p59^fyn^ in later stages of the activation process. T-cells are activated by the antigen receptor followed by a “second signal” provided by the co-receptor CD28 and others (June et al., 1994; Rudd, 1996). In this vein, we showed that p56^lck^ and p59^fyn^ phosphorylate the cytoplasmic tails of CD28 and CTLA-4 (Rudd and Schneider, 2003; Rudd et al., 2009; Figure 3B). They phosphorylate the YxxM sites of both receptors, an event needed for the binding of lipid kinase, phosphoinositide 3-kinases (or phosphatidylinositol 3 kinases; PI 3K), and in the case of CD28, the adaptor complex, GRB-2/Son of Sevenless (SOS) (Prasad et al., 1994; Raab et al., 1995; Schneider et al., 1995a,b). PI 3K, in turn, catalyzes the production of PI-3P from PI and PI 3,4-P2 from PI 4P, a phospholipid that recruits plextrin homology (PH) domain carrying proteins to the plasma membranes. Mutations that affect the levels of PI 3K binding also influences the efficacy of CD28 internalization and removal from the cell surface (Cefai et al., 1998). In this manner, PI 3K is needed for many cellular functions including cell proliferation, endocytosis, differentiation, survival and motility. The p56^lck^ SH3 domain also binds to the p85 subunit of PI 3K thereby bridging of protein tyrosine and lipid kinase pathways in T-cells (Prasad et al., 1993a,b; Kapeller et al., 1994).

The promotion of GRB-2/SOS binding to CD28 by p56^lck^ creates a further link to the p21^ras^ pathway. SOS is a GEF that activates p21^ras^ which, in turn, activates the ERK pathway (Drosten and Barbacid, 2020). p21^ras^ is mutated resulting in a constitutive active protein in 50% of colorectal tumors. In T-cells, to date, GRB-2/SOS complex has been found associated with LAT and CD28. In the case of LAT, it is mediated by ZAP-70 and FAK/PYK2, while the binding to CD28 is mediated by p56^lck^ and p59^fyn^. p56^lck^ and p59^fyn^, therefore, orchestrate the second co-stimulatory step of T-cell activation. This step is followed by CD28 de-phosphorylation needed for the binding of clathrin-linked AP2 complex and endocytosis (Schneider et al., 1999).

Further, CD28 also possesses a more distal key tyrosine which in a phosphorylated form binds to the SH2 domain of p56^lck^ (Kong et al., 2011). The lab of Amnon Altman elegantly showed that the V3 domain of PKC-θ, in turn, binds to CD28 via binding to p56^lck^. Classically, the PKC-θ co-localize and acts as a marker for the central supramolecular signaling cluster (cSMAC) at the center of the interface of T-cells activated with antigen-presenting cells (Shaw and Dustin, 1997; Monks et al., 1998; Freiberg et al., 2002). This pathway implicates CD28 in PKC-θ mediated downstream signaling and the differentiation of T helper type 2 cells (Th2 cells) and interleukin 17-producing helper T cells (Th17 cells), but not of T helper type 1 cells (Th1 cells) (Kong et al., 2011).

## p56^lck^ and Cell Adhesion

Another area involved in the protein-tyrosine phosphorylation cascade involves the “inside-out” pathway by which the antigen-receptor activates integrin adhesion. Adhesion is mediated by LFA-1 and other integrins and is of central importance to T-cell responses. It controls migration within lymph nodes and to sites of infection and mediates binding to antigen-presenting dendritic cells. In this regard, mice with ablated SKAP1 or its binding partner ADAP have normal numbers of T and B-cells, but they are defective in integrin-mediated adhesion (Griffiths et al., 2001; Peterson et al., 2001; Wang et al., 2007, 2009). In the adhesion pathway, SKAP1 is the effector due to its regulation of RapL-Rap1 complex formation (Raab et al., 2010, 2011, 2018). This pathway accounts for some 40–50% of LFA-1 adhesion and contributes to the “slowing” of T-cells for stable interactions with dendritic cells (Wang and Rudd, 2008; Raab et al., 2010).

## p56^lck^ Differs From p59^fyn^

Despite similarities, it is noteworthy that differences exist in the substrates targeted by different p56^lck^ and other SFKs in immune cells. Specifically, p56^lck^ and p59^fyn^ have overlapping and distinct functions. p59^fyn^ can partially substitute for p56^lck^ in T lymphocyte development (Groves et al., 1996) and effector function (Filby et al., 2007); however, p59^fyn^ promotes signals induced by TCR antagonists (Tang et al., 2002) and can inhibit cytokine production and proliferation. Indeed, *p59*^*fyn*−/−^ T-cells are more readily activated, produce more cytokines, and undergo more cell divisions than wild-type T-cells (Filby et al., 2007). Further, unlike p56^lck^, p59^fyn^ only weakly affects Ca^2+^ mobilization, although it can stimulate the ERK/MAPK pathway (Lovatt et al., 2006).

It is not clear how this might be operating, however, importantly, the work from several groups has shown that the two kinases preferentially phosphorylate different substrates. We initially identified ADAP as a preferred substrate and binding partner of p59^fyn^ (hence, it's origin name FYB for Fyn binding protein) (da Silva et al., 1997a,b; Musci et al., 1997; Veale et al., 1999). Kliche and Schraven found that it's binding partner SKAP1 was also preferentially phosphorylated by p59^fyn^ (Marie-Cardine et al., 1997). As mentioned, SKAP1 and ADAP couple the TCR to the activation of integrins (Griffiths et al., 2001; Peterson et al., 2001), while ADAP has an additional role in the activation of the proinflammatory transcription factor, Nfκb (Medeiros et al., 2007). In fact, a mutant of ADAP defective in binding SLP-76 blocks Nfκb driven HIV-1 transcription and cell-cell viral spread (Wei et al., 2013). Lastly, we showed that SKAP1 acts a scaffold for Polo-like kinase 1 (PLK1) for the optimal cell cycling of T-cells (Raab et al., 2019). Whether the differences in p56^lck^ and p59^fyn^ phospho-targets is due to a distinct structural tropism of the kinase domain for different substrates, or simply reflects difference in intracellular localization is unclear. It, therefore, may be that TCR signals bifurcate into a p56^lck^ driven pathways that primarily regulate proliferation and another, p59^fyn^ pathway which preferentially activates integrin mediated adhesion.

## Other Mechanisms for p56^lck^ Function

Despite its importance in signaling in most T-cells, there exists a subset of peripheral T-cell lacking CD4 and CD8 which can be activated via the TCR (D'Acquisto and Crompton, 2011). This begs the question of whether the TCR can also bind to p56^lck^ and whether receptor-free p56^lck^ also plays in role in activation. The unique domain of p56^lck^ has been reported to interact with the CD3ε subunit in the TCR-CD3 complex (Li et al., 2017), while Hartl et al. have reported that non-canonical binding of the lck SH3 domain to the (RK) motif in the CD3ε cytoplasmic tail (Hartl et al., 2020). The RK motif becomes accessible upon TCR ligation, presumably free from interactions with PS molecules in the inner face of the lipid bilayer leading to lck recruitment. This has been reported to increase p56^lck^ activity, CD3 phosphorylation, thymocyte development, and T cell activation (Hartl et al., 2020).

In another model, p56^lck^ unbound to receptors has been found also to play roles in in signaling. Free p56^lck^ was reported by the lab of Nick Gascoigne to be more active than co-receptor bound (Wei et al., 2020). Interestingly, imaging studies showed that free p56^lck^ was recruited to the TCR complex and triggered TCR signaling earlier than the co-receptor-bound p56^lck^ (Nika et al., 2010). The exact temporal nature of involvement of free p56^lck^ relative to co-receptor-bound p56^lck^ in responses of different cells to different affinity ligands needs to be clarified. It may be that some free kinase tweaks the system to then allow CD4 and CD8-p56^lck^ to drive the cascade due to their coordinate interactions with the TCR with the MHC antigens.

## Other Protein Tyrosine Kinases

The notion of a T-cell protein-tyrosine kinase driven phosphorylation cascade led to a flurry of activity to discover other tyrosine kinases and downstream targets in T-cells. It also led to a major effort by pharmaceutical companies to develop kinase specific inhibitors for the treatment of autoimmunity and inflammatory conditions. Aside from the previously mentioned ZAP-70, a second family protein tyrosine kinases termed TEC kinases were uncovered, interleukin 2 inducible T-cell kinase (ITK) and resting lymphocyte kinase (RLK). ITK modulates the development, function and differentiation of conventional T-cells and non-conventional NKT-cells (Schwartzberg et al., 2005). When APCs activate TCR, phosphorylation events lead to the production of D3 lipids and recruitment of ITK to the cell membrane, where it is phosphorylated by p56^lck^. By contrast, unlike p56^lck^, ITK is not needed for CD28 signaling (Li and Berg, 2005). As mentioned, once it is activated, ITK phosphorylates PLCg1 and the mobilization of calcium. ITK operates at later stages of the cascade (Berg et al., 2005) where *Itk*^−/−^ mice fail to mount responses to T_H_2-cell-inducing pathogens. By contrast, mice overexpressing RLK skew differentiation toward the T_H_1-cell lineage. Several studies have also implicated ITK in actin reorganization and cell polarization (Schwartzberg et al., 2005).

Another key family of downstream protein tyrosine kinase includes FAK1 (Focal Adhesion Kinase 1) and PYK2 (proline-rich tyrosine kinase-2). FAKs are comprised of an N-terminal FERM (band 4.1, ezrin, radixin, moesin homology) domain, a linker region, a kinase domain, a large proline-rich region, and a C-terminal focal adhesion targeting domain (Lietha et al., 2007). FAK auto-phosphorylation at the Tyr-397 site is needed for kinase activation and binds to the SH2-domain of p60Src kinase (Arnold et al., 2005). The FERM and kinase domains form an auto-inhibitory interaction (Lietha et al., 2007) which is released in focal adhesions (Arnold et al., 2005). In this context, focal adhesion kinases regulate focal adhesion contacts, motility, and cell survival (Schaller et al., 1992). In T-cells, TCR engagement promotes FAK and PYK2 phosphorylation and translocation to the IS (Sancho et al., 2002; Ostergaard and Lysechko, 2005; Collins et al., 2010). As mentioned, we also recently found that FAK1 and PYK-2 phosphorylate a single specific site on the adaptor LAT for GRB-2 binding and T-cell adhesion (Raab et al., 2017). Non-lymphoid cells from FAK-deficient mice show enhanced focal adhesion contact formation and reduced cell motility (Lietha et al., 2007).

## p56^lck^ and Chimeric Antigen Receptors (CARs)

**Figure 5:**
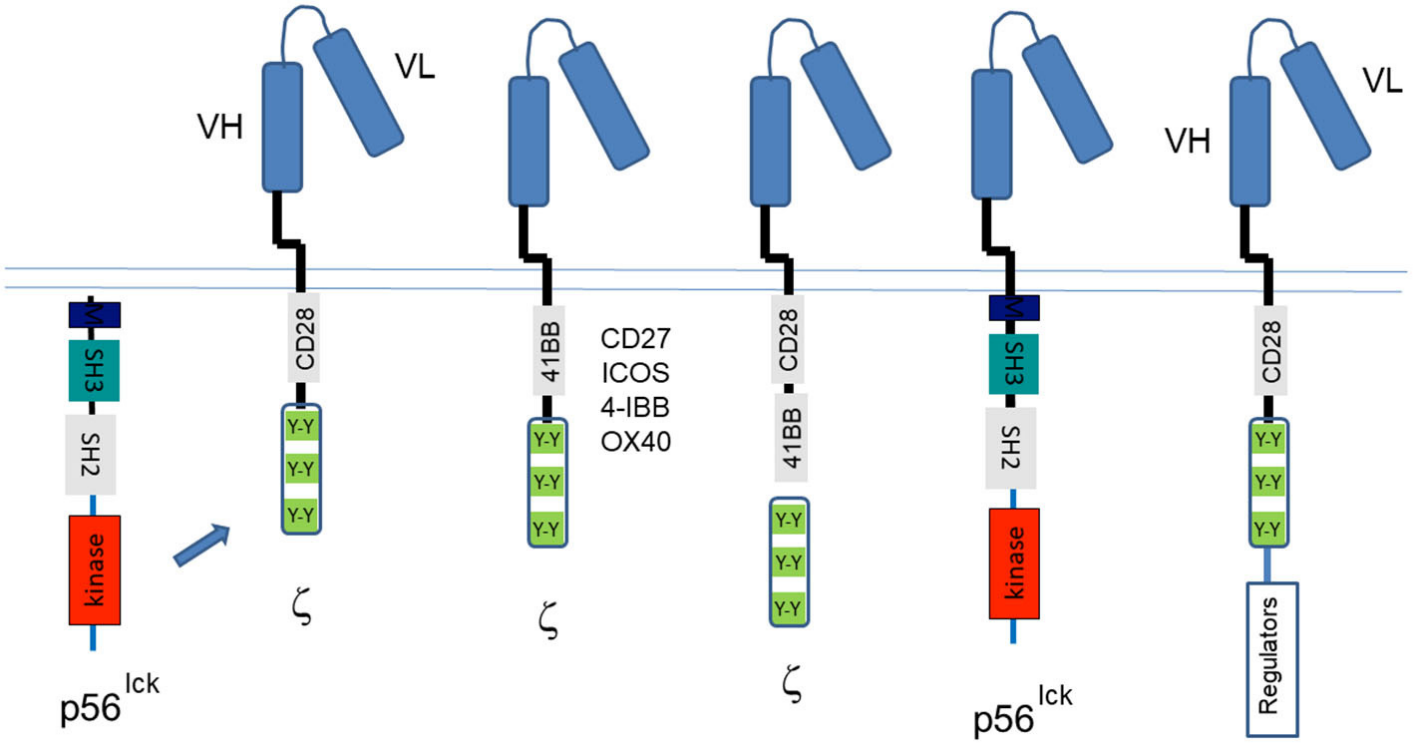
Chimeric antigen receptor (CARs) designed by use of the targets of CD4/CD8-p56^lck^. The discovery of the CD4/CD8-p56^lck^ initiated tyrosine phosphorylation cascade led to the identification of ITAMs and CD28/ICOS/CTLA-4 motifs needed for the activation of T-cells and the preservation of cell survival. The past years have seen many iterations of CARs that began with the Ig ectodomain linked to TCRζ or CD3 ITAMs followed by the inclusion of CD28 cytoplasmic tails (and partial ectodomains). Both the TCRζ and CD3 ITAMs and the CD28 tyrosines are phosphorylated by p56^lck.^ New iterations have included CD27, ICOS, 41-BB, and OX40 motifs in conjunction with ITAMs, dual CD28, and 41BB motifs with ITAMs, the direct coupling to p56^lck^ and the bicistronic inclusion of CD28-ITAMs with the expression of intracellular regulators of metabolism in the tumor microenvironment and in other events in T-cells.

Aside from T-cell activation, the discovery of CD4/CD8-p56^lck^ and its phospho-targets such as ITAMs and CD28 motifs led to the application of this knowledge to the design of chimeric antigen receptors (CARs) (Abate-Daga and Davila, 2016; Kawalekar et al., 2016; Maus and June, 2016). Originally called “T bodies,” almost 30 years ago, by Gross et al. (1989), CARs use antigen-recognition domains derived from an antibody or other proteins that are linked to a transmembrane domain and a intracellular cytoplasmic tail that contains the ITAMs from CD3 or TCR-zeta cytoplasmic tails (Figure 5). The function of these ITAMs is regulated by p56^lck^; however, T-cells expressing first-generation CARs with only ITAMs proved to be short-lived. Instead, additional CD28 “co-signals” were needed to enhance cell survival and *in* anti-tumor killing (June et al., 1994; Rudd, 1996; Finney et al., 1998). As originally seen in the nerve growth factor receptor (Yao and Cooper, 1995), PI-3K to CD28 and CTLA-4 generates survival signals for T-cells (Okkenhaug et al., 2001; Schneider et al., 2008; Rudd et al., 2009). Subsequent variations of CARs contain 4-1BB–derived (Tammana et al., 2010), CD27-derived (Song et al., 2012), OX40-derived (Hombach et al., 2012), or ICOS-derived (Shen et al., 2013) costimulatory sequences. T cells engineered to express CARs with tumor specificity have been remarkable in treating patients with hematologic malignancies in combination with adoptive cell therapy. Their therapeutic success is limited in the case of solid tumors requiring new approaches to address the biology within the tumor microenvironment (TME). To this end, next generation CAR-Ts include bycistronic vectors expressing modulators of the TME. Others have used different exodomain spacers and hinge regions (Watanabe et al., 2016), where the length of the CAR endo-domains determine their ability to interact with endogenous signaling molecules (Ramello et al., 2019). Carl June, a frequent attendee at our signal transduction meetings, has pioneered the use of many CAR-Ts in the treatment of patients (Posey et al., 2016). Some new CAR-Ts are being developed with simultaneous triple genome editing by adding the disruption of PD1 to enhance *in vivo* antitumor activity of the gene-disrupted CAR T cells (Ren et al., 2017). Others have used dual- specific T cells, expressing a CAR specific for tumor antigens, and TCR specific for a strong, tumor-unrelated immunogen (Chan et al., 2020).

Since CARs do not recognize MHC molecules, their reactivity of CAR-Ts is depends on active p56^lck^ to phosphorylate ITAMs and the tyrosine-based motifs within the CD28 co-receptor cytoplasmic tails. However, others have found that the optimal antigen response is dependent upon the incorporation of the receptor in endogenous TCR/CD3 complexes (Bridgeman et al., 2010). These novel approaches may eventually utilize CD4 and CD8 coupled p56^lck^ in addition to free p56^lck^ to promote CAR-T efficacy. Overall, the CAR field developed as a result of fundamental studies that led to the discovery of the TCR complex and the signaling motifs activated by p56^lck^ and which are needed to activate T-cells.

## Summary

The discovery of the CD4 and CD8-p56^lck^ complexes opened a window in understanding the nature of signals that control the immune response against antigens. This fundamental mechanism controls the T-cell response in the areas of vaccines, transplantation, autoimmunity, and cancer. They were the first examples of a receptor binding to protein-tyrosine kinase and showed how immune recognition receptors which lack intrinsic catalytic activity can transduce activation signals via non-covalent association with non-receptor tyrosine kinases. Sometimes called the TCR signaling paradigm, the discovery established that the concept that a protein tyrosine phosphorylation cascade operated in T-cells and opened the door to the identification of other protein-tyrosine kinases such as ZAP-70 and an array of substrates such as immune cell adaptors that are now central to studies in T-cell immunity. Other receptors such as B-cell receptor, Fc receptors and others were also subsequently found to use *src* kinases to control cell growth. Moreover, the discovery of CD4/CD8-p56^lck^ and its targets ITAMs and CD28 has led to the application of this knowledge in the design on CARs presently in use in cancer immunotherapy.

## Author Contributions

The author confirms being the sole contributor of this work and has approved it for publication.

## Conflict of Interest

The author declares that the research was conducted in the absence of any commercial or financial relationships that could be construed as a potential conflict of interest.
